# Scaling Early Detection for Mild Cognitive Impairment at CommonSpirit Health: Leveraging the DAC Blueprint and a Learning Health System Framework

**DOI:** 10.1002/alz70860_100455

**Published:** 2025-12-23

**Authors:** Ankita Sagar, Gary Greensweig

**Affiliations:** ^1^ CommonSpirit Health, Chicago, IL, USA

## Abstract

**Background:**

CommonSpirit Health (CSH), a U.S. faith‐based non‐profit healthcare systems, serves over 20 million patients annually across 24 states through its 2,200+ care sites and 162 hospitals. While CHS has existing initiatives addressing the needs of aging populations, an unmet need persists for timely and accurate detection and diagnosis of Alzheimer's disease and related cognitive impairments. Addressing this challenge is critical given the growing prevalence of cognitive disorders.

**Methods:**

CSH is participating in the Davos Alzheimer's Collaborative Healthcare System Preparedness (DAC‐SP) US Fellowship Project to enhance its capacity to detect mild cognitive impairment. This project builds upon previous initiatives, leveraging established CSH workflows, existing infrastructure, and scaling strategies. The Clinical Standards and Variation Reduction Team (CSVRT), which focuses on accelerating the adoption of evidence‐based medicine across CHS, will implement the DAC‐SP Early Detection Blueprint and develop internal standards to reduce clinical practice variation across CSH's large ambulatory network. Current efforts include site recruitment, early planning, and infrastructure optimization for effective program implementation and scaling.

**Results:**

The DAC‐SP US Fellowship program will enable CSH's efforts to improve cognitive impairment detection and diagnosis by addressing significant variation in current clinical practices. CSVRT will establish a consensus‐driven standard for cognitive screening and follow‐up practices by identifying guidelines, building knowledge, leveraging electronic health records, and sharing data analyses. This Fellowship program will support the optimization of staffing models, utilize technology and human resources effectively, explore reimbursement improvements, and disseminate best practices system‐wide. Early detection practices will be integrated into existing professional development, ensuring sustainable implementation. Programmatic learnings will be shared at the time of presentation.

**Conclusion:**

The DAC‐SP US Fellowship Project serves as a catalyst for CSH's long‐term vision of system‐wide capacity for timely and accurate cognitive impairment detection and diagnosis. Standardized processes, multidisciplinary workflows, and digital innovations, including AI‐enabled remote screening, are central to the strategy. The aim is to create lean, scalable processes that minimize administrative burdens while expanding access to care across diverse geographic regions. By embedding these practices into CSH's operational framework, this Fellowship will ensure sustainable, high‐quality care for cognitive impairment beyond the project's lifespan.

